# The Ferredoxin ThnA3 Negatively Regulates Tetralin Biodegradation Gene Expression *via* ThnY, a Ferredoxin Reductase That Functions as a Regulator of the Catabolic Pathway

**DOI:** 10.1371/journal.pone.0073910

**Published:** 2013-09-12

**Authors:** Laura Ledesma-García, Francisca Reyes-Ramírez, Eduardo Santero

**Affiliations:** 1 Centro Andaluz de Biología del Desarrollo, Universidad Pablo de Olavide, Consejo Superior de Investigaciones Científicas, Junta de Andalucía, Sevilla, Spain; 2 Departamento de Biología Molecular e Ingeniería Bioquímica, Universidad Pablo de Olavide, Sevilla, Spain; Beijing Institute of Microbiology and Epidemiology, China

## Abstract

The genes for tetralin (*thn*) utilization in *Sphingomonas* *macrogolitabida* strain TFA are regulated at the transcriptional level by ThnR, ThnY and ThnA3. ThnR, a LysR-type transcriptional activator activates transcription specifically in response to tetralin, and ThnY is an iron-sulfur flavoprotein that may activate ThnR by protein-protein interaction. ThnA3, a Rieske-type ferredoxin that transfers electrons to the tetralin dioxygenase, prevents transcription of *thn* genes when the inducer molecule of the pathway is a poor substrate for the dioxygenase. The mechanism by which ThnA3 transduces this signal to the regulatory system is a major question concerning *thn* gene regulation. Here, we have confirmed the discriminatory function of ThnA3 and the negative role of its reduced form. We have generated ThnY variants with amino acid exchanges in the [2Fe-2S], FAD and NAD(P) H binding domains and their regulatory properties have been analyzed. Two variants, ThnY-C40S and ThnY-N201G,S206P have completely lost the discriminatory function of the regulatory system because they induced *thn* gene expression with different molecules such us *cis-*decalin, cyclohexane, *trans-*decalin, or benzene, which are not real inducers of the pathway. These results support a model in which ThnA3 exerts its negative modulation *via* the regulator ThnY.

## Introduction

**Figure 1:**
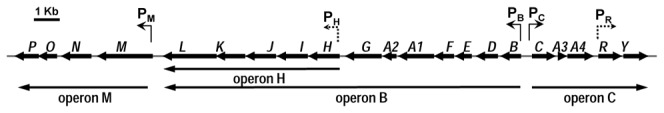
Transcriptional organization of the *thn* genes. Genes are marked by solid arrows, indicating the direction of transcription. Arrows below the genes indicate the 4 main transcriptional units. Bent arrows indicate the identified transcription initiation sites and the direction of transcription. Internal promoters are marked with dashed arrows. *P*_*R*_ *is* a constitutive promoter whilst the others are regulated by tetralin.

Tetralin (1,2,3,4-tetrahydronaphthalene) is a bicyclic molecule composed of an aromatic and an alicyclic moiety, which is found at low concentrations in different crude oils, and it is also industrially produced for its use as an organic solvent. *Sphingomonas* *macrogolitabida* strain TFA is able to use tetralin as a sole source of carbon and energy. The genes coding for the enzymes involved in the tetralin catabolic pathway (*thn* genes) are arranged into four closely linked operons, two of which contain internal promoters (Figure 1). With the exception of the weak constitutive *thnR* promoter, transcription from the *thn* promoters is specifically induced in response to tetralin but is repressed in the presence of preferred carbon sources [1,2]. We have demonstrated by combined genetic and biochemical approaches that three regulatory proteins, named ThnR, ThnY and ThnA3, are the major players involved in *thn* regulation [3,4].

ThnR, the product of the regulatory *thnR* gene, which is co-transcribed together with the structural genes *thnC*, *thnA3* and *thnA4*, encodes a LysR-type transcriptional activator. ThnR activates *thn* gene transcription in response to tetralin by binding to palindromic sites present at each of the four *thn* promoter regions (*P*_*B*_, *P*_*C*_, *P*_*H*_, and *P*_*M*_). Additionally, a mechanism of transcriptional co-regulation of the divergent *thnB* and *thnC* promoters has been reported, which involves the formation of a DNA loop and a higher order structure maintained by interaction between ThnR molecules bound to each promoter region, generating an octameric protein structure [5].

Like ThnR, ThnY is required for *in vivo* transcription of the tetralin biodegradation genes, as *S* *. macrogolitabida* mutants defective in either *thnR* or *thnY* are unable to activate *thn* gene transcription [1,2]. The *thnY* gene presumably is part of the *thnCA3A4RY* operon and its expression is co-regulated in the same manner as all other genes required for tetralin biodegradation [4]. We have recently demonstrated that ThnY shares clear homology to ferredoxin reductases that deliver electrons from NAD(P) H to terminal dioxygenases involved in the degradation of aromatic compounds. Furthermore, purified ThnY has the plant-type [2Fe-2S] cluster and FAD prosthetic groups, which confer similar spectral features reported for structurally related reductases such as benzoate 1,2-dioxygenase reductase [6], naphthalene dioxygenase reductase [7] and carbazole dioxygenase reductase [8]. Despite these similarities, electron transfer through ThnY is not coupled to an obvious catalytic activity, and biochemical analysis clearly demonstrated that ThnY is very inefficient at accepting electrons directly from pyridine nucleotides. In fact, current evidence suggests that ThnY controls the activity of the transcriptional activator ThnR by protein-protein interaction [4].

Regulation of the *thn* genes includes an additional modulation system that discriminates between strong inducers of the pathway, which appear to be metabolized, and weak inducers that are not substrates of the pathway [3]. Mutants lacking either the α (ThnA1) or β (ThnA2) subunits of the tetralin dioxygenase showed very low levels of expression regardless of the inducer. On the other hand, a mutant lacking ThnA3 or the double mutant ThnA1ThnA3 showed high expression levels even with the weakest inducer. ThnA3 is homologous to the Rieske-type ferredoxins that are electron transfer intermediates between the NAD(P) H-dependent ferredoxin reductases and the dioxygenases [9]. Based on these data, it has been proposed that under conditions in which the catabolic pathway cannot efficiently metabolize the inducer molecule, ThnA3 accumulates in its reduced form and prevents induction of tetralin gene expression [3]. This modulation system appears particularly useful for regulating contaminant biodegradation pathways because it represents an efficient way of adjusting the range of potential inducers to the range of metabolizable substrates, therefore avoiding gratuitous induction of a catabolic pathway by similar non-metabolizable molecules. Understanding how the discriminatory function of ThnA3 is transmitted to the regulatory system, ThnR-ThnY, is of critical importance.

Since ThnY is also a modified electron transfer protein, one possibility is that ThnA3 exerts its function by interacting with ThnY. To test this hypothesis, we have generated by site-directed mutagenesis ThnY variants with amino acid replacements located in the [2Fe-2S], FAD and NAD(P) H-binding domains, and the expression phenotype of each of these ThnY variants is presented here. Two variants, ThnY-C40S and ThnY-N201G,S206P showed altered discriminatory capacity of the regulatory system, thus inducing transcription of the *thn* genes with different molecules that are not real inducers of the pathway, such as *cis-*decalin, cyclohexane, *trans-*decalin, or benzene. The *in vivo* data support a model in which the negative modulation of the ferredoxin ThnA3 is exerted on the regulatory system *via* ThnY.

## Results

### ThnA3 negatively modulates *thn* operon expression

In order to characterize the elements involved in regulation of *thn* genes expression further, we have reconstructed the regulation and the modulation mediated by ThnA3 in a more defined genetic background by using the mutant strain T690-690. Strain T690 has a 12.2-kb deletion covering from *thnG* to *thnY* (see Figure 1), which have been replaced by a kanamycin (Km) resistance cassette [10]. As a consequence this strain is unable to grow with tetralin as the sole source of carbon and energy. Besides the 12.2-kb deletion, T690-690 also bears a *thnC::lacZ* translational fusion integrated in its chromosome, which allows measurement of *thn* gene expression [5,10].

Previous experiments had shown that expression of the *thnA1A2A3A4* genes in T690 allowed the initial dioxygenation reaction of the benzene ring of tetralin, since the dioxygenase product, 1,2-dihydroxy-1,2,5,6,7,8-hexahydronaphthalene was produced upon addition of tetralin [10]. We have complemented T690-690 with the isopropyl β-D-thiogalactopyranoside-inducible expression vector pIZ1016 in which we sequentially cloned each of the regulatory elements, *thnR* and *thnY*, in addition to the components of the initial dioxygenase complex, encoding either only the electron transport proteins ThnA3 and ThnA4, or the complete dioxygenase complex ThnA1, ThnA2, ThnA3 and ThnA4.

**Figure 2:**
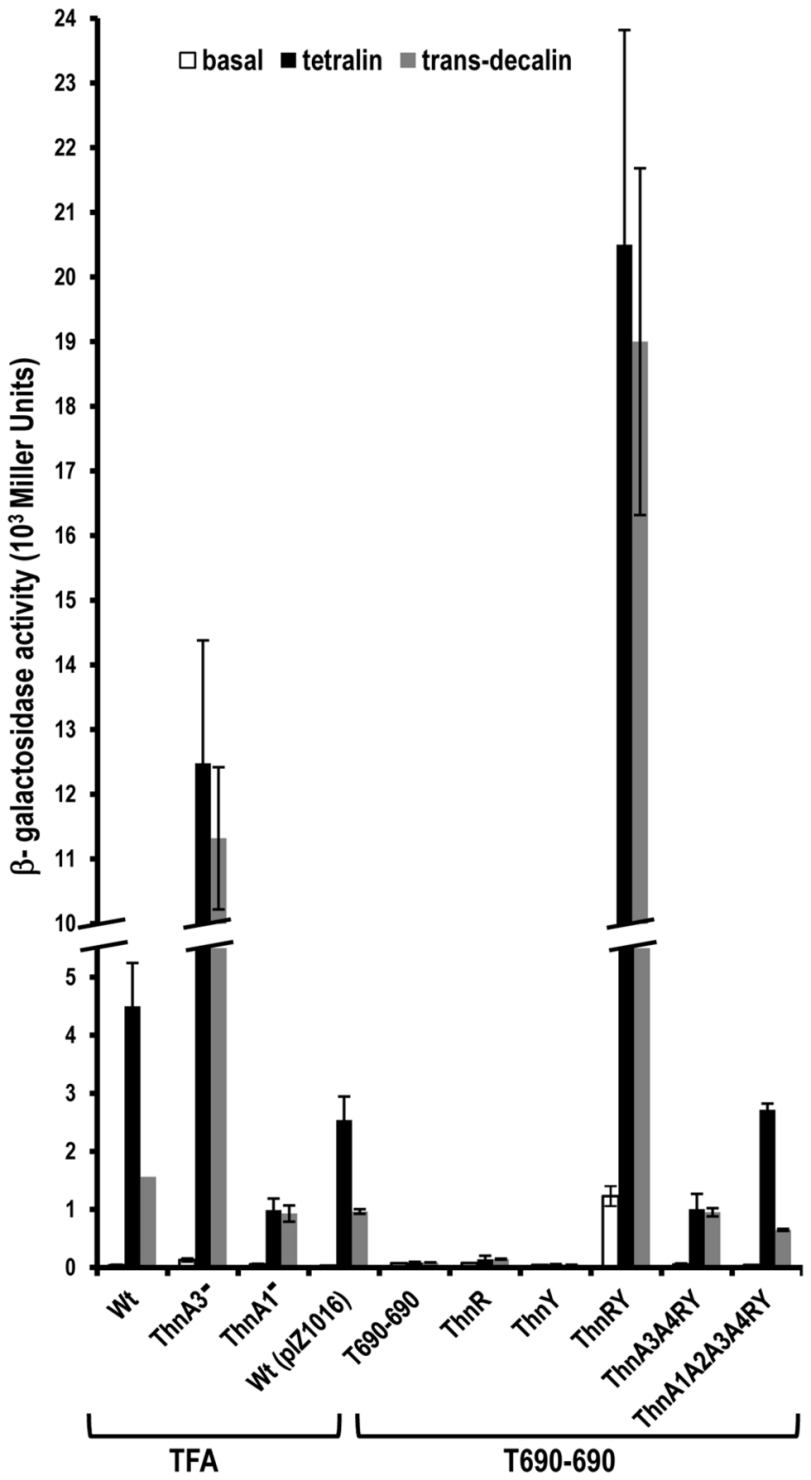
Expression of a *thnC-lacZ* fusion in the T690-690 mutant strain carrying different combinations of the *thnRY* regulatory genes plus the components of the initial dioxygenase *thnA3A4* and *thnA1A2A3A4* from *P*_tac_. β-galactosidase activity was assayed after growth on 8 mM β-hydroxybutyrate (carbon-limiting conditions), in the absence of inducers (white bars), in the presence of tetralin (black bars), or *trans*-decalin (grey bars). Expression levels of the wild type strain without and with the empty vector pIZ1016 are shown. The mutants of TFA lacking the ferredoxin ThnA3 and the α subunit of the dioxygenase (ThnA1) are also included.

To analyse the effect of these proteins on *thn* expression, plasmids pIZ1017 (*thnR*), pMPO750 (*thnY*), pIZ1158 (*thnRY*), pMPO751 (*thnA3A4RY*) and pMPO754 (*thnA1A2A3A4RY*), were introduced into T690-690 and the levels of β-galactosidase activity from the *thnC::lacZ* translational fusion were measured after growth under carbon-limiting conditions with 8 mM of β-hydroxybutyrate (βHB) and in the presence of either tetralin or *trans*-decalin as examples of efficient and inefficient inducers, respectively (Figure 2). The wild type strain, with and without pIZ1016, mutants of TFA lacking the ferredoxin ThnA3 and the α subunit of the dioxygenase (ThnA1) (left part of the figure), and T690-690 under the same inducing conditions were included as controls.

As expected, neither the T690-690 mutant nor its derivative strains expressing *thnR* or *thnY* alone were able to activate expression of *thnC::lacZ*, in agreement with previous reports indicating that both proteins are strictly required for expression of tetralin biodegradation genes [1]. Co-expression of both *thnR* and *thnY* from the *P*_*tac*_ promoter produced very high levels of β-galactosidase activity no matter what molecule was used to induce the system, indicating that under these circumstances the regulatory system, though responsive to the effector molecules, had completely lost the ability to discriminate between them. This is essentially the same expression phenotype observed for mutant strains lacking the ferredoxin ThnA3 [3]

To analyse the effect of adding ThnA3 on *thn* gene expression, T690-690 was complemented with plasmid pMPO751, which carries genes encoding ThnA3 and the ferredoxin reductase ThnA4, its electron donor partner, in addition to the regulatory proteins ThnR and ThnY. As shown in Figure 2, induction of *thn* gene expression by either tetralin or *trans*-decalin was clearly reduced. These results are in complete agreement with the expression phenotype of *thnA1* mutants, which lack dioxygenase activity, in which presumably an intracellular accumulation of reduced ThnA3 maintains *thn* gene expression at a very low level even in the presence of tetralin [3]. When all the components required for initial dioxygenation of tetralin (*thnA1A2A3A4*) were included, in addition to the regulatory genes, the wild type expression profile, i.e. high induction levels using tetralin and low levels using *trans*-decalin, was completely restored (Figure 2).

Taken together, these results identify the elements that influence the levels of *thn* gene expression and clearly indicate that the ferredoxin ThnA3 has a discriminatory role among potential inducers, preventing transcription under conditions that presumably lead to accumulation of the reduced form of the protein. This situation may be found in the wild type strain in the absence of a suitable substrate for the dioxygenase (complementation with pMPO754 with *trans*-decalin), or in a mutant lacking tetralin dioxygenase (complementation with pMPO751 even with tetralin). In summary, this finding confirms the discriminatory function of ThnA3 and the negative role of its reduced form.

### Construction of *thnY* mutants altered in electron transport cofactors

As previously reported, ThnY does not contain all the properties of its ferredoxin reductase homologs, but it possesses functional [2Fe-2S] cluster and FAD prosthetic groups, as they are able to undergo reduction in the presence of sodium dithionite [4]. Thus, one possibility is that negative modulation by the reduced form of ThnA3 is exerted through redox changes in ThnY, which in turn, would affect its function in activating *thn* gene expression.

**Figure 3:**
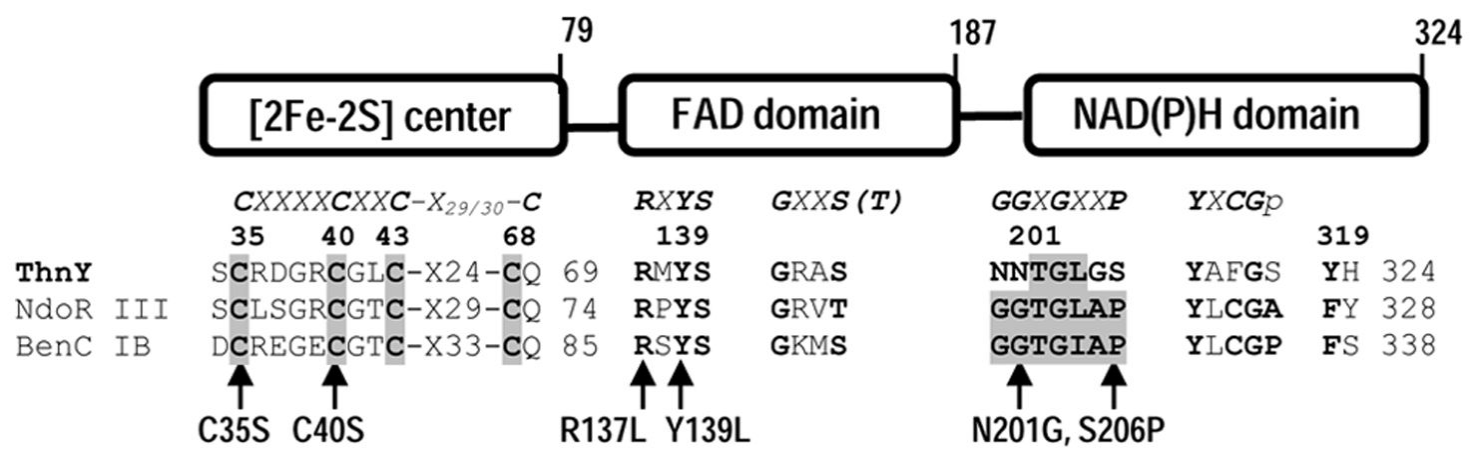
Diagrammatic representation of the three-domain structure of ferredoxin reductase representatives of Classes IB and III of dioxygenases systems. BenC, benzoate 1,2-dioxygenase reductase from *Acinetobacter* sp. [11] and NdoR, naphthalene dioxygenase reductase from *Pseudomonas* [25]. Conserved sequences for the three cofactors binding domains of those reductases and the identified conserved motifs in ThnY are shown. The conserved cysteines for plant-type [2Fe-2S] cluster are shaded and in bold; conserved residues for binding of isoalloxazine and phosphate groups of the FAD, respectively, are shown in bold. The highly conserved glycine fingerprints involved in binding of NAD(P) H in plant/bacterial-type FNRs (ferredoxin:NAD(P)^+^ reductases) are shaded; other conserved amino acid and the aromatic residue involved in the interaction with the adenine moiety of FAD are also indicated in bold [4]. Amino acid replacements characterized in this study are indicated by arrows.

In order to investigate whether the electron transfer properties of ThnY are important for its function as a regulatory protein, and to establish a relation between its structure and function, codons encoding conserved amino acid residues critical for binding of the prosthetic groups were mutated by site-directed mutagenesis, as detailed in Materials and Methods. Residues Cys^35^ and Cys^40^ of ThnY, corresponding to the highly conserved residues C-X4-C-X2-C-X29/30-C for plant-type [2Fe-2S] cluster binding, were substituted by serine, a structural analog of cysteine. Amino acids Arg^137^ and Tyr^139^ of ThnY, corresponding to the highly conserved residues RXYS are of particular importance for binding of the isoalloxazine ring of the flavin [11], and therefore these residues were exchanged for Leu, an amino acid with an aliphatic side chain. These changes should disrupt the hydrogen-bonding network to the FAD. Although the NAD(P) H-binding domain of ThnY is still recognizable, amino acid sequence alignment at the position of the glycine signature (GGXGXXP), proposed to be involved in the binding of the adenosine-5´-phosphate groups of NADH or 2´-phospho-AMP of NADPH [12], showed that the first two glycines are occupied by asparagines (N200,N201) and the conserved proline is replaced by a serine (S206) [4]. Given that a deletion of the whole NAD(P) H domain resulted in non-functional ThnY (not shown), suggesting that it might have a role controlling the regulatory function of ThnY, the corresponding N201 and S206 residues of ThnY were changed to the highly conserved glycine and proline, respectively. Figure 3 shows a diagrammatic representation of the domain structure and consensus peptide segments involved in the plant-type [2Fe-2S] cluster, flavin and NAD(P) H-binding motifs in representative ferredoxin reductases of dioxygenases systems [9], where the locations of the mutations constructed in this study are also indicated.

### Effect of *thnY* mutations on the regulation of *thn* expression

**Figure 4:**
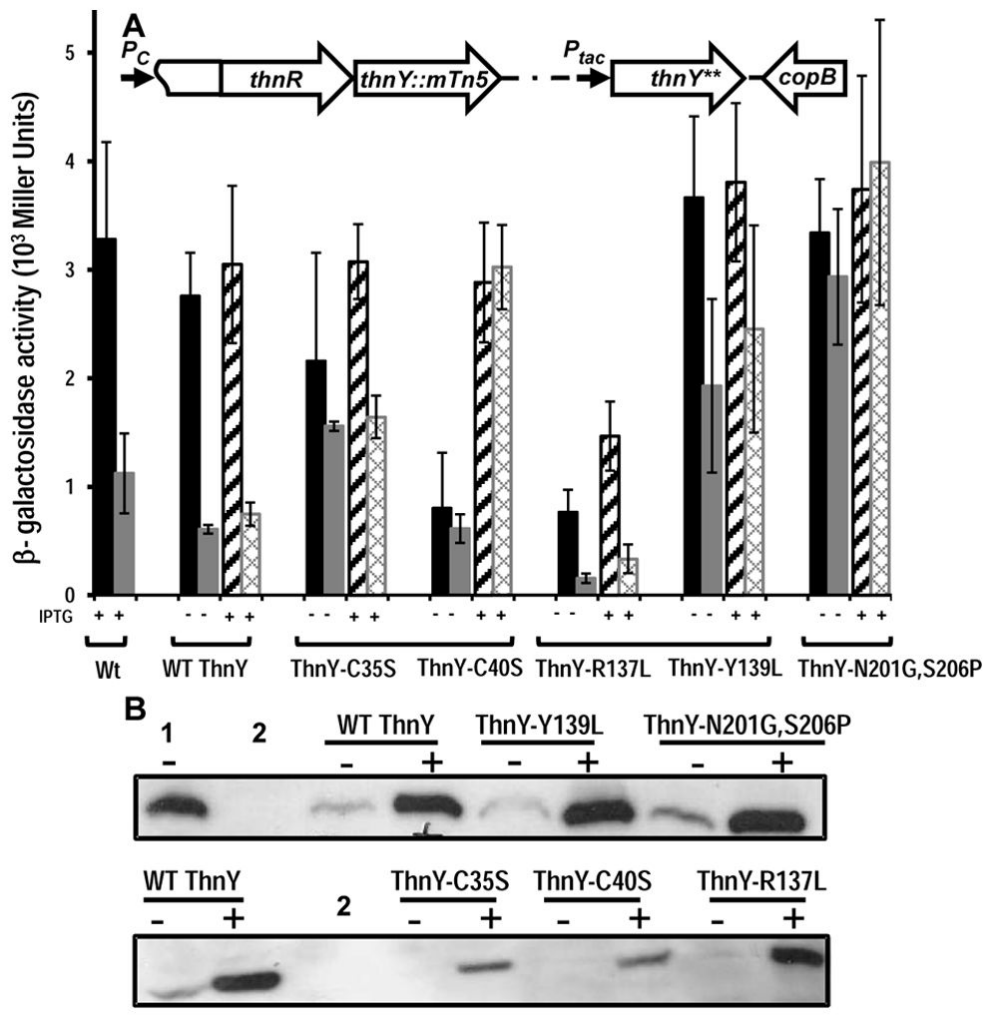
Effect of ThnY variants on *thn* gene expression. (A) Cultures were grown under carbon-limiting conditions using the following conditions: tetralin (black bars); *trans*-decalin (grey bars); tetralin plus IPTG (black hatched bars); and *trans*-decalin plus IPTG (grey cross-hatched bars). β-galactosidase activity was measured as described in Materials and Methods. Values represent the means of at least three independent determinations. The following plasmids expressing either wild type or ThnY variants were used for complementation of mutant strain T601-1002: pMPO750 (wild-type ThnY), pMPO764 (ThnY-C35S), pMPO765 (ThnY-C40S), pMPO757 (ThnY-R137L), pMPO756 (ThnY-Y139L), pMPO763 (ThnY-N201G,S206P). (+) and (-) indicate addition or not of IPTG. A schematic representation of the *thnRY* region after the recombination event leading to the integration of the *thnY* derivatives into the chromosome is represented on the top. thnY** denotes WT *thnY* or its mutant derivatives. (B) Western blot analysis showing the level of ThnY accumulated in the tetralin-induced cultures used for the β-galactosidase assays with (+) and without (-) IPTG. This allows direct comparison of the accumulated levels of ThnY since the amount of total protein in each lane is the same. ThnY synthesis in the wild type TFA, (1), and in T601–1002, (2), was included for comparison.

To examine the *in vivo* effect of the ThnY amino acid variants on transcriptional activation by ThnR, the T601-1002 strain, which carries a mini-Tn*5*km inserted into codon 140 of *thnY* and a *thnC::lacZ* translational fusion [1] also inserted into the chromosome, was complemented with the pIZ1016 derivative plasmids pMPO750, expressing wild type *thnY* and pMPO764, pMPO765, pMPO757, pMPO756 and pMPO763, expressing the mutant derivatives C35S, C40S, R137L, Y139L and N201G,S206P, respectively. These constructs were designed to place the expression of *thnY* alleles under the control of the *P*_*tac*_ promoter. All plasmids resulting from the inducible expression vector pIZ1016 tend to integrate into the TFA chromosome, introducing the mutated DNA regions into its chromosome (data not shown). Therefore, those colonies that had placed the *thnY* alleles under the control of the *P*_*tac*_ promoter after the first recombination event were selected by PCR. A schematic representation of the final genome organization around the *thnR* and *thnY* region is shown in Figure 4A. The effect of these ThnY variants on *thn* gene expression, as well as their capacity of distinguishing between a good and a bad substrate of the catabolic pathway, was determined under carbon-limiting conditions using either tetralin or *trans*-decalin as inducers and with or without IPTG, as indicated in Figure 4A. The levels of the respective ThnY protein variants in each strain and condition are also shown in Figure 4B. T601-1002 was unable to grow with tetralin as the sole source of carbon and energy and to induce expression of the *thnC-lacZ* fusion (data not shown). Plasmid pMPO750, expressing WT ThnY, complemented the *thnY* mutation because it allowed the mutant strain to grow on tetralin as the only carbon and energy source (not shown), and restored the levels and expression profile to those of the wild type TFA strain (Figure 4A). The *thnC-lacZ* expression levels were similar regardless of whether IPTG was added, even though the concentration of ThnY in the absence of IPTG was very low, much lower than that produced by the wild type TFA strain (Figure 4B). Thus, it appears that the ThnY concentration even in the absence of IPTG was sufficient to obtain maximal induction levels (Figure 4).

The absolute induction levels using ThnY-R137L were clearly lower than those using WT ThnY. This low level is consistent with the slower growth of the strain and reduced total biomass in the presence of tetralin (not shown), as compared to the WT ThnY. Western blots showed that production of ThnY-R137L was approximately similar to that of the WT ThnY (Figure 4B), therefore, the lower expression levels using ThnY-R137L indicate that this protein was partially active. These results suggest that the R137 of ThnY plays an important role in *thn* transcriptional activation. However, the ThnY-R137L protein variant produced an induction profile similar to that of the WT ThnY, i.e. higher induction by tetralin than that by *trans*-decalin, thus indicating a discrimination capacity similar to that of WT ThnY.

Mutants ThnY-C35S and ThnY-Y139L performed as the wild type protein, since both were able to restore growth on tetralin (not shown) and gave similar levels of *thn* gene expression (Figure 4A). This indicates that their functionality was not substantially affected by the amino acid exchanges. However, they appear to be slightly affected in discrimination capacity between strong and weak inducers. For example, induction by *trans*-decalin was always higher than 50% of the induction by tetralin, as compared to the 25% that was obtained using WT ThnY. On the other hand, although the mutation *thnY-N201G,S206P* also did not affect the functionality of ThnY, the protein variant had clearly lost its discrimination capacity, since the expression levels induced by *trans*-decalin were very similar to those caused by tetralin (88% without and 106% with IPTG). As shown by the Western blots (Figure 4B) and the expression levels (Figure 4A) increasing the amount of ThnY-Y139L or ThnY-N201G,S206P by addition of IPTG did not result in a substantial increase in the levels of *thn* gene expression in the presence of tetralin, suggesting that, as in the case of the WT ThnY, the low concentration of these proteins even in the absence of IPTG was also saturating.

The ThnY-C40S variant had also lost its discrimination capacity between tetralin and *trans*-decalin either in the absence (induction by *trans*-decalin was 76% of that by tetralin) or in the presence of IPTG (induction by *trans*-decalin was 104% of that by tetralin) (Figure 4A). In this case, augmenting the amounts of ThnY-C40S by adding IPTG increased induction by tetralin to completely restore the wild type expression levels, thus indicating that ThnY function was not saturating in the absence of IPTG. Since accumulation of this protein variant was significantly lower than the WT ThnY (Figure 4B), we interpret this to mean that the lower induction of gene expression in the absence of IPTG does not indicate a limited functionality of ThnY-C40S but is rather due to a very low level of ThnY-C40S, which is undetectable under this condition.

Altogether the results presented here indicated that alterations in the redox cofactor binding domains of ThnY are not important for the activation function but rather are critical for the discriminatory function of the regulatory system. This effect is strongest in the case of the C40S and the double N201G,S206P amino acid substitutions since they have completely lost the ability to discriminate between tetralin and *trans*-decalin therefore activating the catabolic pathway under inappropriate conditions.

**Figure 5:**
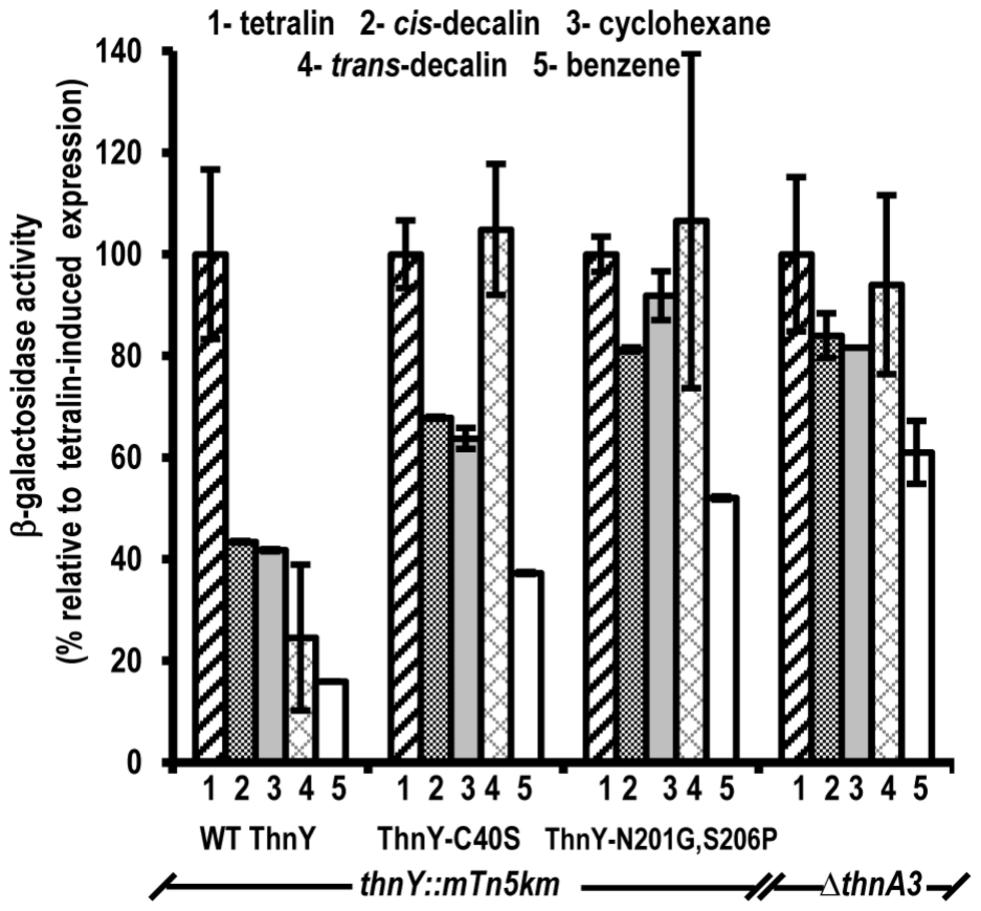
Inducer profile for *thn* operon expression in ThnY-C40S and ThnY N201G,S206P variants. The relative efficiency of different molecules to induce expression was measured by quantifying the levels of expression of a *thnC::lacZ* fusion under carbon limiting conditions containing IPTG and the indicated molecules as inducers. For each protein variant, induction obtained with tetralin was set to 100%. The *ΔthnA3* strain under the same growth conditions is also included for comparison.

In order to confirm whether these two ThnY variants had altered the capacity to discriminate between different inducer molecules, we extended the analysis by examining induction by other molecules containing aromatic or alicyclic rings (Figure 5). As mentioned above for *trans*-decalin, complementation of the chromosomal *thnY* mutant strain (T601–1002) with WT *thnY* clearly showed that these molecules were obviously less efficient inducers than tetralin (40% for *cis*-decalin and cyclohexane, and 15% for benzene, numbers 2, 3 and 5 respectively in Figure 5). These data agree with those already observed with the wild type TFA [3]. In contrast, in the mutant strains expressing *thnY-C40S* and *thnY-N201G,S206P*, molecules such as *cis*-decalin and cyclohexane induced the tetralin biodegradation genes to levels substantially higher than those obtained with the WT *thnY*, and more similar to those obtained with tetralin, the true substrate of the pathway, (68-65% for the mutant ThnY-C40S and 81%-92% for the double mutant, respectively).

As shown in Figure 5, this expression phenotype resembles that of mutant strains lacking the ferredoxin ThnA3, because they are able to activate the catabolic pathway to high levels in response to compounds other than tetralin. Overall, these results confirm that the C40S and N201G,S206P substitutions diminish the efficiency of the discriminatory response of the *thn* system.

### Effect of the ferredoxin ThnA3 on the activity of ThnY-C40S and ThnY-N201G,S206P

It is clear from the results shown above that the variants ThnY-C40S and ThnY N201G,S206P cannot distinguish between different molecules, suggesting that they might escape the negative modulation exerted by the ferredoxin ThnA3. Therefore, we decided to examine the activities of these mutants in a strain lacking ThnA3 and in a strain presumably accumulating the ferredoxin ThnA3 in its reduced state, as is the case with the *thnA1* point mutant*, thnA1-D221A*. The amino acid substitution Asp221Ala renders the tetralin dioxygenase fully inactive by apparently preventing electron transfer from the ferredoxin to the catalytic center and, consequently, to the substrate [13,14].

**Figure 6:**
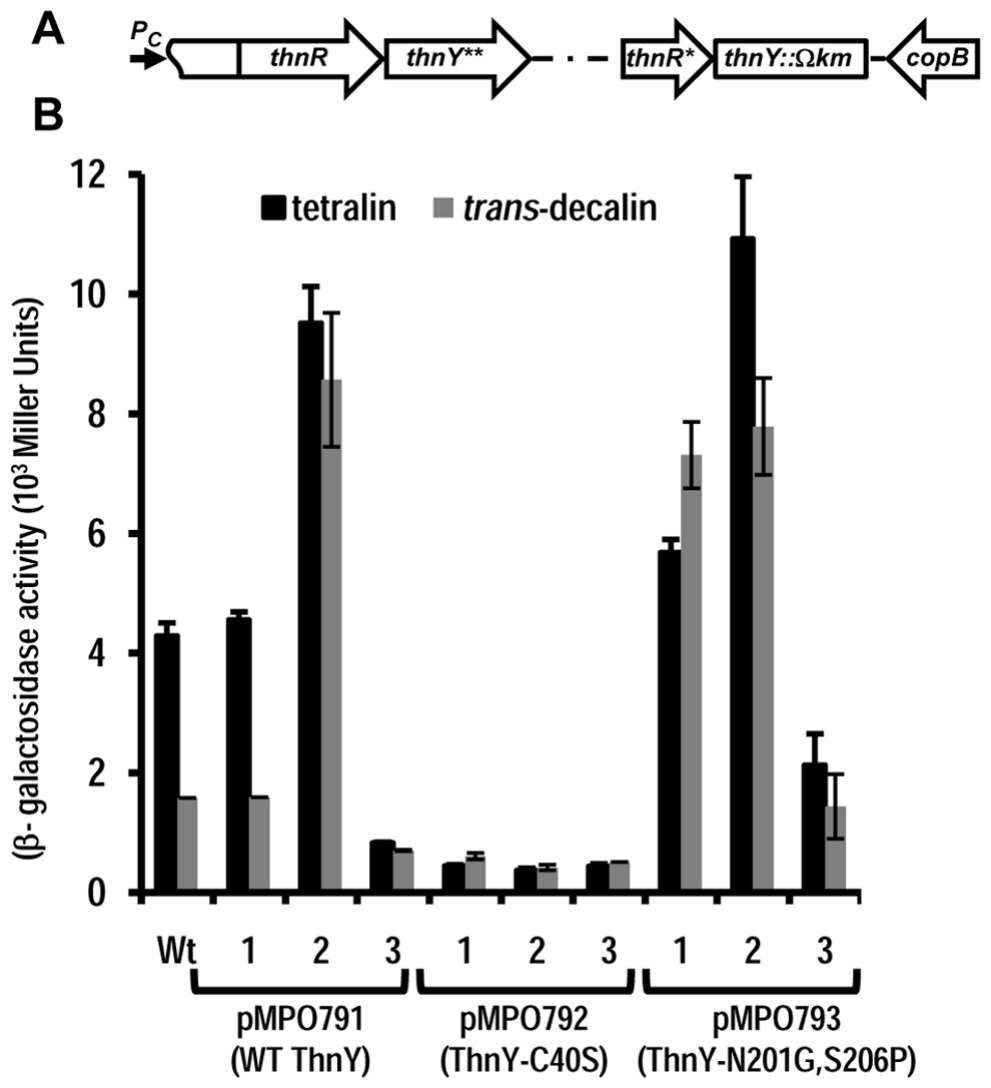
Influence of ThnA3 on the ability of ThnY-C40S and ThnY-N201G,S206P to induce *thn* expression. (A) Schematic representation of the genome organization of the *thnRY* region after the recombination event leading to integration of the *thnY* derivatives into the chromosome. thnY** denotes WT *thnY* or its mutant derivatives, while thnR* indicates a deletion of 243 pb from its 5’ end. Arrows indicate the positions and directions of the genes. As shown in the schematic, the *P*_*c*_ promoter drives transcription of *thnR* and *thnY*. (B) Ability of ThnY-C40S and ThnY-N201G,S206P to induce *thn* expression in *thnY* :*:Ωkm* (1), *thnY*:*:Ωkm∆thnA3* (2) and the *thnY*:*:ΩkmthnA1-D221A* (3) mutants strains. Plasmids pMPO791 (wild type *thnY*), pMPO792 (thnY-C40S) and pMPO793 (*thnY*-*N201G*,*S206P*) were used for integration. Cultures were assayed for β-galatosidase activity from a *thnC::lacZ* reporter fusion after growth under carbon-limiting conditions using tetralin (black bars) and *trans*-decalin (grey bars) as inducers.

To that end, the double mutant strains *thnYthnA3*, and *thnYthnA1* were generated and complemented with derivatives of pIZ1016 expressing the WT *thnY* or the mutant alleles, *thnY-C40S* and *thnY*-*N201G*,*S206P*. In order to analyze these mutations in the natural genomic context, we integrated them into the bacterial chromosome. Therefore, we had to construct a new chromosomal version of the *thnY* mutation in which the entire gene was replaced by a Km resistance Ω cassette, strain *thnY*:*:Ωkm* (MPO751), to avoid recombination between *thnY* sequences. The double-mutant strains, *thnY*:*:Ωkm*∆*thnA3* (MPO752) and the *thnY*:*:ΩkmthnA1-D221A* (MPO753), were accordingly constructed based on this background strain. For complementation studies, appropriate DNA fragments containing either the wild type *thnY* allele, the *thnY-C40S* or the *thnY-N201G*,*S206P* derivatives were re-cloned into a promoter-less derivative of pIZ1016, which has just 675 bp of the *thnR* 3' end and the whole *thnY*-coding sequence. Theses constructs were designed to place the *thnY* alleles under the control of the chromosomal *Pc* promoter. A schematic representation of the genome organization around the *thnR* and *thnY* region after the recombination event leading to the integration of the *thnY* derivatives is shown in the Figure 6A.

To study the expression phenotype, the levels of β-galactosidase activity from the *thnC::lacZ* translational fusion, which was integrated in each strain, was measured after cells were grown with 8 mM of βHB (carbon-limiting conditions) in the presence of either tetralin or *trans*-decalin. Figure 6B shows that complementation with the WT *thnY* gene clearly reproduced the expected expression phenotype: (i) complementation of *thnY*:*:Ωkm* (strain number 1 in the figure) resulted in lower induced levels with *trans*-decalin than with tetralin; (ii) the levels of *thn* gene induction were very high in the absence of ThnA3, no matter the inducer molecule, (*thnY*:*:Ωkm∆thnA3*, strain number 2); and (iii) induction was very low in the genetic background in which reduced ThnA3 is presumably abundant, (*thnY*:*:ΩkmthnA1-D221A*, strain number 3), regardless of the presence of the inducer. In contrast, ThnY-C40S exhibited the same low level of expression regardless of the inducer molecule, and the genetic background. The absence of ThnA3 (strain 2) or its presence mainly in the reduced form (strain 3) did not substantially affect the expression levels, thus indicating that this mutation renders the protein fully insensitive to the negative effect of the ferredoxin. These results clearly indicate that the information as to whether a molecule is an appropriate inducer or not is transduced from ThnA3 to the regulatory system *via* the ferredoxin reductase ThnY.

Complementation of the *thnY*:*:Ωkm* mutant (strain 1 in Figure 6) with *thnY-N201G,S206P* reproduced what has been shown above in Figures 4A and 5: the *thnY-N201G,S206P* mutation conferred the inability to discriminate between a weak and a strong inducer. However, tetralin induction increased in the absence of ThnA3 (strain 2) and expression was clearly limited when ThnA3 could not reduce the dioxygenase enzyme (strain 3), regardless of the inducer. These data clearly suggest that ThnY-N201G,S206P is somehow still sensitive to the negative effect of the ferredoxin, although this mutant form does not allow discrimination between the inducers.

## Discussion

Based on the phenotype of mutants affected in each component of the initial tetralin dioxygenase enzymatic complex, which catalyzes the first reaction, we proposed a model in which ThnA3, an intermediate ferredoxin that transfers electrons to the tetralin dioxygenase, plays a role in this signal transduction [3]. Involvement of ThnA3 in *thn* regulation *in vivo* is confirmed in this study (i) by reproducing tetralin regulation in a genetic background deleted for all *thn* genes, to which different factors involved were sequentially added (Figure 2), and (ii) by complementing *thnY thnA1* and *thnY thnA3* double mutants with wild type or mutant *thnY* alleles (Figure 6B). In all cases, results were consistent with our working model and showed that ThnR and ThnY activate the *thn* promoters promiscuously, whilst the ferredoxin ThnA3 prevents transcription activation when the inducer molecule is not the real substrate of the pathway. Therefore, ThnA3 enables the bacterium to discriminate between an efficient substrate for the catabolic pathway and a non-metabolizable molecule, thus preventing gratuitous induction by a non-substrate molecule, even though it had been recognized as an inducer molecule by the regulatory system (Figures 2 and 5). Although many Fe–S cluster proteins have been reported to also have regulatory functions [15], to our knowledge, this is the first time that an intermediate dioxygenase ferredoxin has a role in regulating transcription.

Once confirmed the regulatory role of ThnA3, the key question is how it exerts its function. There are several reasons that prompted us to think of ThnY as a potential signal transduction partner for ThnA3: (i) ThnY is essential for transcription activation of the *thn* genes [1,3] and increase ThnR specific DNA-binding probably *via* protein-protein interaction [4]; (ii) ThnY is a modified ferredoxin reductase that can be reduced by sodium dithionite [4]. These data suggested that ThnY activity may be redox-sensitive, and that ThnA3 could therefore control ThnY function by reducing it. Here we have generated a number of ThnY variants, two of which clearly have altered its regulatory properties, because they allowed the bacterium to induce tetralin degradation genes in response to *trans*-decalin, *cis*-decalin or cyclohexane, molecules that do not contain aromatic rings and, therefore, cannot be substrates for the pathway (Figure 5). As these mutants have impaired the discriminatory capacity, they provide genetic evidence indicating that ThnY forms part of the signal transduction pathway that prevents *thn* gene induction in the absence of a metabolizable substrate.

The phenotype that most obviously supports this view is that exhibited by the mutant ThnY-C40S, in the [2Fe-2S] cluster. The interesting features of this mutant are: (i) it has completely lost the capacity to discriminate between molecules that are substrates for dioxygenation or not (Figures 4 and 5); and (ii) it clearly escaped the negative regulatory effect of ThnA3, since ThnY-C40S activity is exactly the same in a strain lacking the ferredoxin as in a strain in which the terminal dioxygenase is not functional (Figure 6B). Thus, it appears that ThnY-C40S is locked in its ‘on’ state because it is fully insensitive to the inhibitory function of ThnA3. The phenotype of the ThnY-N201G,S206P mutant appears to be different from that of ThnY-C40S, and has a more complex interpretation, since these substitutions rendered a ThnY variant defective in discrimination but it still retained sensitivity to ThnA3 (Figure 6B).

**Figure 7:**
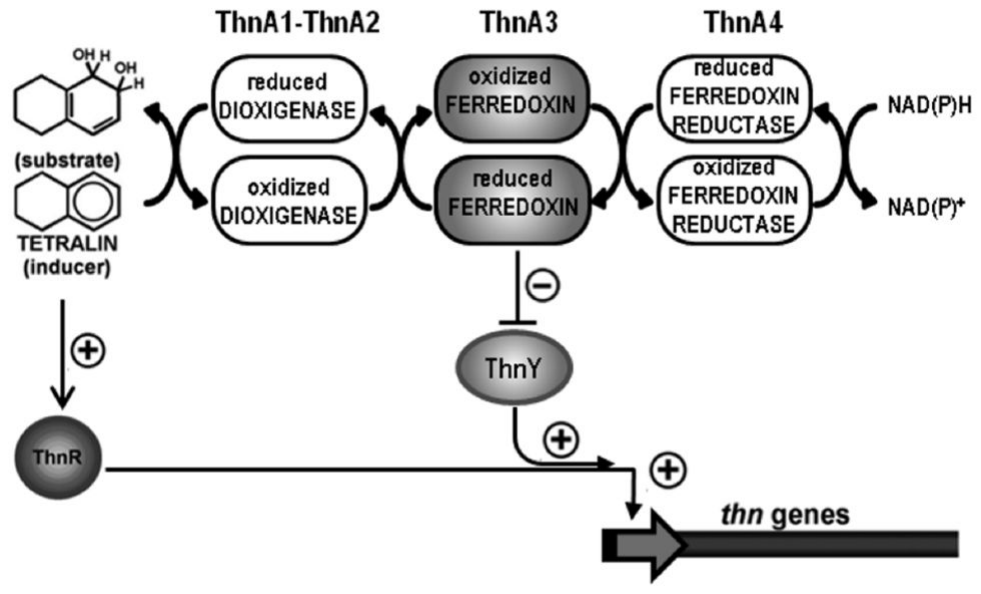
Current model for regulation of tetralin degradation genes. Model showing that the negative role of the reduced form of ThnA3 in response to a non-metabolizable molecule of the pathway is exerted *via* the co-activator ThnY.

Figure 7 shows our current model for signal transduction regulating *thn* expression in response to potential inducer molecules. Considering that ThnA3 limits the induction potential of ThnR-ThnY in the wild type strain, even when tetralin is the inducer (Figure 2), we propose that when ThnA3 is essentially in its oxidized form it still partially inactivates ThnY. However, this inhibition is maximal when ThnA3 is predominantly in its reduced form, i.e., when the inducer is not a substrate for the pathway, presumably by reducing ThnY. Of course, in the dioxygenase mutants, i.e. *thnA1* mutants, all ThnA3 is fully available to interact with ThnY, thus the regulatory system becomes more efficiently inactivated regardless of the inducer. In other words, the ability to discriminate between a gratuitous inducer and tetralin comes from the different capacity of ThnA3 to inactivate the regulatory system, depending on its redox state and its subsequent reduction of ThnY, which would be maximal in the absence of a substrate. According to this view, the phenotype of the ThnY-N201G,S206P mutant can be explained if this variant form is still able to interact with ThnA3 and remains sensitive to the binary complex formed with ThnA3, but cannot be reduced by ThnA3. Therefore, this form of ThnY does not allow discrimination between inducer molecules as a function of the reduced state of ThnA3.

## Materials and Methods

### Media and bacteria growth conditions

*E. coli* strains were routinely grown in Luria-Bertani medium at 37 °C; *S* *. macrogolitabida* strains were grown at 30 °C in MML rich medium [16] or MM medium [17] with β-hydroxybutyrate as the carbon and energy source.

Antibiotics were used, when were required, at the following final concentrations: ampicillin (100 µg/ml or 5 µg/ml for *E. coli* or *S* *. macrogolitabida* strains respectively); kanamycin (20 µg/ml for *E. coli* or 10 µg/ml S*. macrogolitabida* strains); gentamicin (10 µg/ml); and streptomycin (50 µg/ml for *S* *. macrogolitabida* strains).

### Construction of plasmids and bacterial strains

Strains, plasmids and primers used in this work are summarized in the Table S1. Plasmid pMPO751 bearing *thnA3A4RY* under the *P*_*tac*_ promoter, was constructed by insertion of a 3.37 kb *Pvu*II-*Hind*III DNA fragment from pIZ612 into pMPO750 digested with *Sal*I(Klenow) +*Hind*III.

A 3.35 kb *Hpa*I-*Xba*I DNA fragment bearing *thnRY* from pMPO751, was introduced into pIZ652 digested with the same enzymes used to generate pMPO753, which carries an artificial *thnA1A2A3A4RY* operon. This plasmid was used to create pMPO754. The 6.5 kb *Xho*I-*Xba*I DNA fragment bearing the *thnA1A2A3A4RY* operon from pMPO753 was inserted into the broad-host-range expression vector pIZ1016 digested with *Sal*I-*Xba*I to obtain pMPO754 in which *thnA1A2A3A4RY* was transcribed from the *P*_*tac*_ promoter.

Plasmids pMPO756, pMPO757, pMPO763, pMPO764 and pMPO765 carry different mutant *thnY* alleles under the control of the *P*_*tac*_ promoter were generated by overlapping PCR [18]. To do that, two internal and divergent primers introducing the appropriate mutations were used together with the external primers, and pMPO750 as the template. The two resulting PCR products were mixed in 1:1 and amplified with the external primers to obtain a single DNA segment containing the mutations. Those DNA fragments were cloned into pMPO750 to substitute the *thnY* wild type region.

pMPO756, which contains the Tyr139Leu (TAT to TTA) substitution in the FAD domain of *thnY*, was constructed by using thnY-YL1 and thnY-YL2 as internal primers, and SalI-orFY12 and thnY2-1Q as the external ones, respectively. The final 1 kb DNA fragment was digested with *Sal*I+*Pst*I and the 0.49 kb fragment was introduced into pMPO750 digested with the same enzymes.

pMPO757, which contains the Arg137Leu (CGC to CTC) substitution in the FAD domain of *thnY*, was constructed essentially as pMPO756 but using thnY-RL1 and thnY-RL2 as internal primers. The final PCR fragment was digested and cloned into pMPO750 in the same way that pMPO757.

pMPO763, which contains the Asn201Gly (AAT to GGT) and Ser206Pro (TCG to CCG) substitutions in the NAD(P) H domain of ThnY, was constructed essentially as above but using thnY-NG-SP1 and thnY-NG-SP2 as internal primers. The final 1 kb DNA fragment was digested with *Sal*I+*Hind*III, and a 0.84 kb DNA fragment was cloned into pMPO750 digested with the same enzymes.

pMPO764, which contains the Cys35Ser (TGC to AGC) substitution in the fer domain of *thnY*, was executed by using orFY3 and orFY4 as internal primers, and SalI-orFY12 and orFY10 as the external ones, respectively. The final 0.56 kb DNA fragment was digested with *Sal*I+*Pst*I and the 0.49 kb fragment was introduced into pMPO750 digested with the same enzymes.

pMPO765, containing the Cys40Ser (TGC to AGC) substitution in the [2Fe-2S] domain of *thnY*, was done in the same way that pMPO764 but using orfY7 and orFY8 as internal primers. The final PCR fragment was digested and introduced into pMPO750 as in pMPO764.

Plasmids pMPO791, pMPO792 and pMPO793 harbored the different *thnY* versions (WT *thnY*, *thnY-C40S* and *thnY-N201G,S206P* respectively) downstream a fragment of 675 pb of the C-terminal region of *thnR* under no promoter control.

pMPO791, with the wild type *thnY* was build digesting pIZ1158 with *Sal*I+*Pst*I. The 1.1 kb DNA fragment was cloned into pMPO750 digested with *Nco*I (T4 endonuclease) +*Sal*I.

pMPO792, which carries the *thnY-C40S* mutant was done by substitution of the 0.38 kb DNA fragment bearing the mutation of pMPO765 digested with *Bsa*I+*Pst*I, for the same DNA fragment of pMPO791 digested in the same way.

pMPO793 was construct essentially as pMPO791, but the DNA fragment from pIZ1158 digested with *Sal*I+*Pst*I was cloned into pMPO763 *Nco*I(T4 endonuclease) +*Sal*I.

pIZ1159 was made to generate a chromosomal deletion mutation of *thnY*. The DNA fragment was made by overlapping PCR using pIZ1158 as template and orFY1+orFY6 as primers to amplify the upstream fragment, and orFY2+orFY5 to amplify the downstream one. Primers orFY1 and orFY2 incorporated an *EcoR*I restriction site, while orFY5 and orFY6 a *Sac*I one. The resultant PCR products were mixed in 1:1, and amplified with the external orFY1 and orFY2 primers. The final 2.45 kb DNA fragment carrying *thnRcopB* was digested with *EcoR*I and cloned into *EcoR*I-digested Bluescript II SK+ to produce pIZ1157. The *Ωkm* fragment was obtained by treating pMKm with *EcoR*I and Klenow, and cloned into the generated *Sac*I site to construct pIZ1159.

The *thnY*:*:Ωkm*, (*ΔthnY*) mutant MPO751 was obtained by marker exchange replacement of wild type *thnY* with the *Ωkm* fragment present in pIZ1159. A first recombination event leading to integration of pIZ1159 was selected as kanamycin and ampicillin resistant clones. The second recombination event leading to *thnY* substitution was obtained as ampicillin sensitive kanamycin resistant clones. The MPO752 (*thnY*:*:*Ω*km*,*ΔthnA3* double mutant) and MPO753 (*thnY*:*:*Ω*km*,*thnA1-D221A* double mutant) strains were obtained in a similar way by marker exchange replacement of *thnY* in the mutant T1031 (*ΔthnA3*) and T1034 (*thnA1*’) strains, with the *Ωkm* of pIZ1159. The authenticity of all of the strains constructed by marker exchange was confirmed by Southern blot hybridation.

To monitor the expression levels of the *thn* genes by β-galactosidase assays, plasmids carrying *thnC::lacZ* gene fusions (in pMPO690 for the *Δthn* mutant strain T690, or in pIZ1002 in all the other cases) were introduced by triparental mating and integrated by homologous recombination.

For complementation analysis, the different plasmids were transferred into the recipient strains and transconjugant colonies were selected on MML agar plates by using resistance to gentamicin, ampicillin, kanamycin and streptomycin. Incorporation of the plasmids and, eventually, their integration into the chromosome, were verified by PCR.

All DNA manipulations were performed according to standard procedures [19]. DNA plasmids were transferred into *E. coli* and *S* *. macrogolitabida* strains by transformation [20] or triparental mating [21] respectively. *E. coli* DH5α was used as host in all cloning procedures.

### Induction assays

Induction assays of *S* *. macrogolitabida* strains containing *lacZ* fusions integrated into the chromosome were performed as previously described [1]. The inducers tetralin, cyclohexane, *cis*-decalin, *trans*-decalin and benzene were supplied in the gas phase. When required, IPTG was used at 1 mM final concentration. Cultures were grown for 22 h at 30 °C in minimal medium containing 8 mM βHB as the carbon source, and β-galactosidase activity was assayed as described by Miller [22].

### Western Blot Analysis with anti-TFA ThnY antiserum

Whole-cell protein extracts from each TFA strain were prepared from tetralin-induced cultures used for the β-galactosidase assays with (+) and without (-) IPTG. To do that, 8 ml of cultures were harvesting and the cell pellets were resuspended in an appropriate volume of buffer that was adjusted according to differences in optical density at 600 nm. To calculate the concentration of protein and to further ensure that biomass among samples were equivalent, several dilutions in water from these samples were prepared in duplicates and the total amount of protein in each sample was determined by the method of Lowry [23] using bovine serum albumin as the standard. Aliquots of these suspensions were mixed with reducing 2× SDS sample buffer (160 mM Tris-HCl [pH 6.8], 4% [wt/vol] SDS, 20% [vol/vol] glycerol, 0.1% [wt/vol] bromophenol blue, 10% [vol/vol] 2-mercaptoethanol), boiled for 10 min, and centrifuged for 1 min. Extracts corresponding to 250 µg of total protein per sample were run on 12% (w/v) SDS-PAGE polyacrylamide gels [24]. For Western blotting, rabbit anti-ThnY polyclonal antibodies [4] was diluted 5000-fold before use, and the antibody–antigen reaction was visualized using SuperSignal Western Blotting kit (*Thermo Scientific*) according to the manufacturer’s specifications.

## Supporting Information

Table S1 **Strains, plasmids and primers used in this work.**
(DOCX)
