# Supplementary material for: The Ferredoxin ThnA3 Negatively Regulates Tetralin Biodegradation Gene Expression via ThnY, a Ferredoxin Reductase That Functions as a Regulator of the Catabolic Pathway
Source: PLoS One. 2013 Sep 12;8(9):e73910. doi: 10.1371/journal.pone.0073910 (PMC3771892; doi:10.1371/journal.pone.0073910)
Supplement: Table S1 — Strains, plasmids and primers used in this work. (DOCX) [file pone.0073910.s001.docx]

| **Bacterial strains** | **Relevant genotype/phenotype** | **Origin/Reference** |
| --- | --- | --- |
| ***E. coli*** DH5α | [F^-^ φ80d *lacZ*ΔM15 Δ(*lacZYA*–*argF)U169recA1 endA1 hsdR17* (r_k_^-^m_k_^-^) *supE44 thi-1gypA relA1*] | *[26]* |
| ***S. macrogolitabida*** | | |
| TFA | wild type strain. Str^r^ | *[27]* |
| T601 | *thnY*::*mTn5km*, inserted in *Pst*I, codon 140. Str^r^, Km^r^ | *[1]* |
| T601-1002 | T601mutant with *thnC::lacZ* translational fusion into the chomosome. Str^r^, Ap^r^, Km^r^ | *[1]* |
| T690 | *Δthn*, substitution of 12.2 kb of the C and B *thn* operons by a KIXX insertion. Str^r^, Km^r^ | *[10]* |
| T690-690 | *Δthn*, wild type *thnB-thnC* intergenic region. *thnC::lacZ* translational fusion. Str^r^, Ap^r^, Km^r^ | *[5]* |
| T1031 | *ΔthnA3* mutant. Str^r^ | *[3]* |
| T1034 | *thnA1-D221A* mutant (*thnA1´*). Str^r^ | *[3]* |
| MPO751 | *thnY::Ωkm* mutant *(ΔthnY)*. Str^r^, Km^r^ | *This work* |
| MPO751-1002 | *thnY::Ωkm *mutant with *thnC::lacZ* translational fusion into the chomosome. Str^r^, Ap^r^, Km^r^. | *This work* |
| MPO752 | *thnY::ΩkmthnA3 *double mutant* thnYΔthnA3)*. Str^r^, Km^r^ | *This work* |
| MPO752-1002 | *thnY::ΩkmthnA3* double mutant with *thnC::lacZ* translational fusion into the chomosome. Str^r^, Ap^r^, Km^r^ | *This work* |
| MPO753 | *thnY::Ωkm,thnA1-D221A *double mutant *(ΔthnYthnA1´)*. Str^r^, Km^r^ | *This work* |
| MPO753-1002 | *thnY::ΩkmthnA1D221A* double mutantwith *thnC::lacZ* translational fusion into the chomosome. Str^r^, Ap^r^, Km^r^ | *This work* |
| MPO785-1002 | MPO751(*ΔthnY*)-1002mutant withplasmid pMPO791 integrated into the chromosome. WT *thnY* under *P_C_*_._ Str^r^, Ap^r^, Km^r^, Gm^r^ | *This work* |
| MPO786-1002 | MPO751(*ΔthnY*)-1002 mutant withplasmid pMPO792 integrated into the chromosome. *thnY*-*C40S* under *P_C_*. Str^r^, Ap^r^, Km^r^, Gm^r^ | *This work* |
| MPO787-1002 | MPO751(*ΔthnY*)-1002mutant with plasmid pMPO793 integrated into the chromosome. *thnY*-*N201G,S206P* under *P_C_*. Str^r^, Ap^r^, Km^r^, Gm^r^ | *This work* |
| MPO788-1002 | MPO752(*ΔthnYΔthnA3*)-1002mutant withplasmid pMPO791 integrated into the chromosome. WT *thnY* under *P_C_*. Str^r^, Ap^r^, Km^r^, Gm^r^ | *This work* |
| MPO789-1002 | MPO752(*ΔthnYΔthnA3*)-1002 mutant withplasmid pMPO792 integrated into the chromosome. *thnY*-*C40S* under *P_C_*. Str^r^, Ap^r^, Km^r^, Gm^r^ | *This work* |
| MPO790-1002 | MPO752(*ΔthnYΔthnA3*)-1002 mutant with plasmid pMPO793 integrated into the chromosome. *thnY*-*N201G,S206P* under *P_C_*. Str^r^, Ap^r^, Km^r^, Gm^r^ | *This work* |
| MPO791-1002 | MPO753(*ΔthnYthnA1´*)-1002 mutant withplasmid pMPO791 integrated into the chromosome. Wt *thnY* under *P_C_*. Str^r^, Ap^r^, Km^r^, Gm^r^ | *This work* |
| MPO792-1002 | MPO753(*ΔthnY thnA1´*)-1002mutant with plasmid pMPO792 integrated into the chromosome. *thnY*-*C40S* under *P_C_.* Str^r^, Ap^r^, Km^r^, Gm^r^ | *This work* |
| MPO793-1002 | MPO753(*ΔthnY thnA1´*)-1002mutant withplasmid pMPO793 integrated into the chromosome. *thnY*-*N201G,S206P* under *P_C_*. Str^r^, Ap^r^, Km^r^, Gm^r^ | *This work* |
| **Plasmids** | | |
| pBluescript II SK/KS+ | Cloning vector. Ap^r^ | *Stratagene* |
| pIZ612 | *P_T7_- thnBCA3A4RY´* into pTZ18U. Ap^r^ | *[27]* |
| pIZ652 | *P_T7_- thnA1A2A3A4* into Bluescript II SK+. Ap^r^ | *[28]* |
| pIZ698 | *P_tac_- thnY* *cop´B* into pIZ1016. Gm^r^ | *[1]* |
| pIZ1002 | *thnC::lacZ* translational fusion into pJES379. Ap^r^ | *[1]* |
| pIZ1016 | pBBRMCS-5 broad-host-range-vector derivative, with the *tac* promoter and *lacl*^q^ from pMM40. Gm^r^ | *[1]* |
| pIZ1017 | *thnR* in pIZ1016, Gm^r^ | *[1]* |
| pIZ1157 | *P_T7_- thnRcop´B* into Bluescript II SK+. Ap^r^, Km^r^ | *This work* |
| pIZ1158 | *P_tac_- thnRYcop´B* into pIZ1016. Gm^r^ | *[5]* |
| pIZ1159 | *P_T7_- thnRΩkmcop´B* into Bluescript II SK+. Ap^r^, Km^r^ | *This work* |
| pMKm | *aphA* gene | *[29]* |
| pMPO690 | pIZ1002 derivative bearing the KIXX cassette from pUC4KIXX in *EcoR*I. Ap^r^, Km^r^ | *[5]* |
| pMPO750 | *P_tac_- thnY* wild type into pIZ1016. Gm^r^ | *This work* |
| pMPO751 | *P_tac_- thnA3A4RY* into pIZ1016.Gm^r^ | *This work* |
| pMPO753 | *P_T7_- thnA1A2A3A4RY* into Bluescript II SK+. Ap^r^ | *This work* |
| pMPO754 | *P_tac_- thnA1A2A3A4RY* into pIZ1016.Gm^r^ | *This work* |
| pMPO756 | *P_tac_- thnY*-*Y139L* mutant into pIZ1016. Gm^r^ | *This work* |
| pMPO757 | *P_tac_- thnY*-*R137L* mutant into pIZ1016. Gm^r^ | *This work* |
| pMPO763 | *P_tac_- thnY*-*N201G,S206P* mutant into pIZ1016. Gm^r^ | *This work* |
| pMPO764 | *P_tac_ -thnY*-*C35S* mutant into pIZ1016. Gm^r^ | *This work* |
| pMPO765 | *P_tac_- thnY*-*C40S* mutant into pIZ1016. Gm^r^ | *This work* |
| pMPO791 | *thn’RY* without any promoter into pIZ1016. Gm^r^ | *This work* |
| pMPO792 | *thn’RY*-*C40S* without any promoter into pIZ1016. Gm^r^ | *This work* |
| pMPO793 | *thn’RY*-*N201G,S206P* without any promoter into pIZ1016. Gm^r^ | *This work* |
| pRK2013 | helper plasmid for conjugation. Km^r^, Tra^+^ | *[30]* |
| **Primers** | **Sequence 5´- 3´** | **Reference** |
| SalI- orFY12 | CCCGTCGACCCAATGTGTGGCTGAGG | *This work* |
| thnY2-1Q | CGCTACGCCCTTGTTTCG | *López-Sánchez (unpublished)* |
| thnY- YL1 | GCCTGCTGCAGATAACATGCG | *This work* |
| thnY- YL2 | CGCGCATGTTATCTGCAGCAA | *This work* |
| thnY- RL1 | GCAGAATACATGAGCGTGATG | *This work* |
| thnY- RL2 | CGACATCACGCTCATGTATTC | *This work* |
| thnY- NG- SP1 | CGGGCCAAGTCCCGTACCGTTGGATATGCACAGG | *This work* |
| thnY- NG- SP2 | GGTACGGGACTTGGCCCGTTGCTCGGCTTCC | *This work* |
| orFY1 | GGAATTCAGGAGTGGAAGCTGGAGGAAGTGCGGAC | *This work* |
| orFY2 | GGAATTCCGGTGATTGATTGGGAATTGGATGCCGTAAG | *This work* |
| orFY3 | CCATCCCTGCTACTGTGCGAAATAGG | *This work* |
| orFY4 | CCTATTTCGCACAGTAGCAGGGATGG | *This work* |
| orFY5 | GGCTTGAGCTCTGGCGCACTAATCAGACGGTTTGGAG | *This work* |
| orFY6 | CCAGAGCTCAAGCCGGCCCCAATTGAGCAGTTG | *This work* |
| orFY7 | AAAGGCCGCTCCGGCCATCCCTGCAACGT | *This work* |
| orFY8 | GGATGGCCGGAGCGGCTTTTGCAAATGTTC | *This work* |
| orFY10 | CCGATGCCCGACCGAGATTGTGTAG | *This work* |

**REFERENCES**

26. Hanahan D (1983) Studies on transformation of Escherichia coli with plasmids. J Mol Biol 166: 557-580.

27. Hernaez MJ, Reineke W, Santero E (1999) Genetic analysis of biodegradation of tetralin by a Sphingomonas strain. Appl Environ Microbiol 65: 1806-1810.

28. Royo JL, Moreno-Ruiz E, Cebolla A, Santero E (2005) Stable long-term indigo production by overexpression of dioxygenase genes using a chromosomal integrated cascade expression circuit. J Biotechnol 116: 113-124.

29. Murillo J, Shen H, Gerhold D, Sharma A, Cooksey DA, et al. (1994) Characterization of pPT23B, the plasmid involved in syringolide production by Pseudomonas syringae pv. tomato PT23. Plasmid 31: 275-287.

30. Figurski DH, Helinski DR (1979) Replication of an origin-containing derivative of plasmid RK2 dependent on a plasmid function provided in trans. Proc Natl Acad Sci U S A 76: 1648-1652.
